# Brain Tumor Classification Using AFM in Combination with Data Mining Techniques

**DOI:** 10.1155/2013/176519

**Published:** 2013-08-25

**Authors:** Marlene Huml, René Silye, Gerald Zauner, Stephan Hutterer, Kurt Schilcher

**Affiliations:** ^1^School of Applied Health and Social Sciences, University of Applied Sciences Upper Austria, Garnisonstraße 21, 4020 Linz, Austria; ^2^Department of Pathology, Nerve Clinic Linz Wagner Jauregg, Wagner-Jauregg-Weg 15, 4020 Linz, Austria; ^3^University of Applied Sciences Upper Austria, Research & Development Wels, Stelzhamerstraße 23, 4600 Wels, Austria

## Abstract

Although classification of astrocytic tumors is standardized by the WHO grading system, which is mainly based on microscopy-derived, histomorphological features, there is great interobserver variability. The main causes are thought to be the complexity of morphological details varying from tumor to tumor and from patient to patient, variations in the technical histopathological procedures like staining protocols, and finally the individual experience of the diagnosing pathologist. Thus, to raise astrocytoma grading to a more objective standard, this paper proposes a methodology based on atomic force microscopy (AFM) derived images made from histopathological samples in combination with data mining techniques. By comparing AFM images with corresponding light microscopy images of the same area, the progressive formation of cavities due to cell necrosis was identified as a typical morphological marker for a computer-assisted analysis. Using genetic programming as a tool for feature analysis, a best model was created that achieved 94.74% classification accuracy in distinguishing grade II tumors from grade IV ones. While utilizing modern image analysis techniques, AFM may become an important tool in astrocytic tumor diagnosis. By this way patients suffering from grade II tumors are identified unambiguously, having a less risk for malignant transformation. They would benefit from early adjuvant therapies.

## 1. Introduction

Following the classification of the World Health Organization (WHO) astrocytic tumors (gliomas) are divided into four grades, which are typically assigned on the microscopic appearance of the tumor [1]. Grade I comprises pilocytic astrocytoma, and grades II to IV represent invasive tumors having progressive malignancy and worse prognosis. Grade I gliomas are mainly localized respecting anatomic boundaries, whereas grades II to IV gliomas are infiltrating the tissue at different extents [2, 3]. This characteristic makes an exact localization and an accurate determination of the grade by surgical biopsy difficult [4]. To reach more efficacy noninvasive imaging techniques are used. Computed tomography (CT) scanning, magnetic resonance imaging (MRI), positron emission tomography (PET) scanning, and a lot of advanced MR techniques enhance the ability to localize the tumor and to determine the grading enormously [5]; nevertheless in some individual cases there is noticeable disagreement in clinical diagnosis. The reason for this may be mainly attributed to great interobserver variability [6]. Every pathologist assesses individually each of the grading criteria defined in the WHO grading scheme, based on the subjective evaluation of the tumor. So an objectification based on statistically derived features independent from subjective analysis would be an important additional element in astrocytoma grading.

Atomic force microscopy (AFM) has become a widely used technique for characterizing biological samples at nanometer resolution. In a lot of studies [7–16] AFM has been successfully used to image living brain cells under physiological conditions. An imaging of native brain tissue, however, has not been done so far because of the elastic compliance of the soft brain tissue which reduces imaging quality. A few studies [17–19] have been performed using histological brain slices. Different types of brain cells, some of their organelles, and the neuropil were recognizable at the tissue level. 

A common method to decide grading of astrocytoma is the examination of H&E-stained histological brain slices with a light microscope [5, 20] using characteristic histopathological features, such as hypercellularity, distinct vascular proliferation, mitotic activity, grade of pleomorphism, and necrosis [21]. This method, however, strongly depends on the staining protocol itself and the quality of the used optics of the examining microscopes. Slight variations in the thickness of the slices, differences in fixation time, or differences in the chemical purity of the used staining reagents influence the quality of the images. A robust and objective way to analyse histological samples like astrocytoma neuropathological slides would offer AFM. AFM requires no staining, which has the additional advantage that the samples are not masked by any chemistry. Due to the fact that AFM scan size is limited (100 × 100 *μ*m^2^), overview images cannot be acquired, so special large-scaled histopathological features like vascular proliferation or number of mitosis are hard to observe. Small-scaled phenomena like pleomorphism or the local formation of cavities due to neuropil reduction, however, may be observed very well and could serve as additional features to the conservative grading method. 

Accurate classification of brain tumor grading is very important in the diagnosis because it defines prognosis and treatment decision for the patient. Dependant on the used standard imaging technique many refined methods were developed to increase grading accuracy [22–24]. All these methods have lack of imaging brain tissue down to the cellular level, and thus in addition a histological examination of biopsied or resected tumor tissue is always being performed. Both techniques, imaging and histology, use special data mining methods [6, 25–36]. Besides morphology, a lot of recent investigations have specialized in molecular techniques, specially in the analysis of gene expression profiles [37]. They correlate with clinical outcome and in some cases predict a better survival than histological classification [38, 39].

In this study, we present an objective method, which is well suited to enhance the accuracy in the determination of specific tumor features. The presented method differs from other ones by the combination of two key elements: (a) high-resolution microscopy where we will show that AFM imaging on histological unstained brain samples is able to deduce relevant morphological information, which can be used for grading astrocytoma; (b) image analysis where we will demonstrate that the application of special data mining algorithms based on Minkowski functionals enables an objective, automatic identification of histomorphological features also in such a complex task like astrocytoma grading. This automatic approach enables improved classification accuracy in the future and could offer new diagnostic elements for an objectivized morphological tumor categorization.

## 2. Material and Methods

### 2.1. Sample Preparation

The clinical material comprised brain tumor samples from 14 patients that were made available from the pathological institute of the Nerve Clinic Wagner-Jauregg in Linz (Austria). The samples were classified as low (astrocytoma grade II, *n* = 7) and high (glioblastoma multiforme grade IV, *n* = 7) brain tumors according to the WHO grading system by experienced histopathologists. They represent a small selection of typical patterns and do not fully represent the diagnostic category. 

The samples were prepared according to standard pathology protocol [19]. In short, the histological preparation contained the following steps: (1) fixation in 4% formaldehyde in phosphate buffer saline (PBS), (2) dehydration with ethanol and embedding in paraffin, (3) sectioning in 3 *μ*m slices using a microtome (Leica RM 2155), (4) deparaffining by submerging in xylol and following treating with ethanol and (5) conservation in distilled water. Before the measurements took place, all samples were washed with distilled water to remove salt residues which would produce artifacts on the images. Additionally, slices from the same paraffin block (patient) were stained with haematoxylin and eosin (HE-staining). These specimens were used as a reference for assigning the grading of the tumor and for defining regions of interest (ROI) in a light microscope prior to AFM scanning. 

### 2.2. Microscopy

#### 2.2.1. Atomic Force Microscopy

The AFM measurements were performed on an Agilent 5400 AFM/SPM (Agilent Technologies Inc., Santa Clara, CA, USA), equipped with a large multipurpose scanner and a digital camera (Navitar Inc., Rochester, NY, USA). All images were acquired in air at room temperature in contact mode using commercial non-conductive silicon nitride cantilevers (Bruker Corporation, Camarillo, CA, USA) with a spring constant between 0.005–0.06 N/m. The images were taken at 512 × 512 pixels quality at a scanning rate of 1.0 lines/second. All images were recorded with PicoView 1.4.2 (Agilent Technologies Inc., Chandler AZ, USA) and further processed with Pico Image Basic 5.0.4.5170 (Agilent Technologies Inc., Chandler, AZ, USA). Altogether the analysis comprised images of 113 samples (54 astrocytoma grade II and 59 glioblastoma multiforme grade IV). 

#### 2.2.2. Light Microscopy

Light microscopy was performed on a Nikon eclipse ME 600 (Nikon Instruments Austria, Vienna). The magnifications were 50, 100, and 500.

### 2.3. Image Analysis

**Figure 1:**
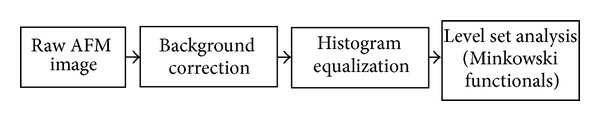
The main processing steps in image analysis.

**Figure 2:**
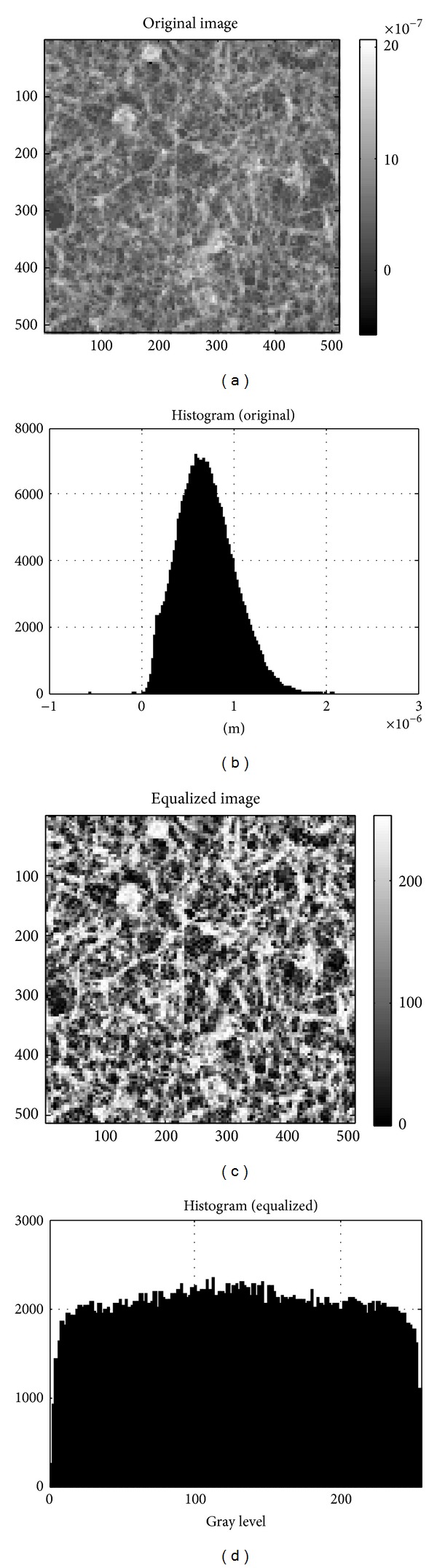
Histogram equalization for a typical astrocytoma grade II AFM image. (a) Original AFM image composed of 512 × 512 pixels. The corresponding scan size was 100 × 100 *μ*m^2^. The vertical bar shows differences in height in m. (b) Height histogram of the original data. (c) Equalized AFM image after the transformation step spreading out the height level histogram to a full dynamic range of 256 gray levels. (d) Corresponding equalized histogram.

**Figure 3:**
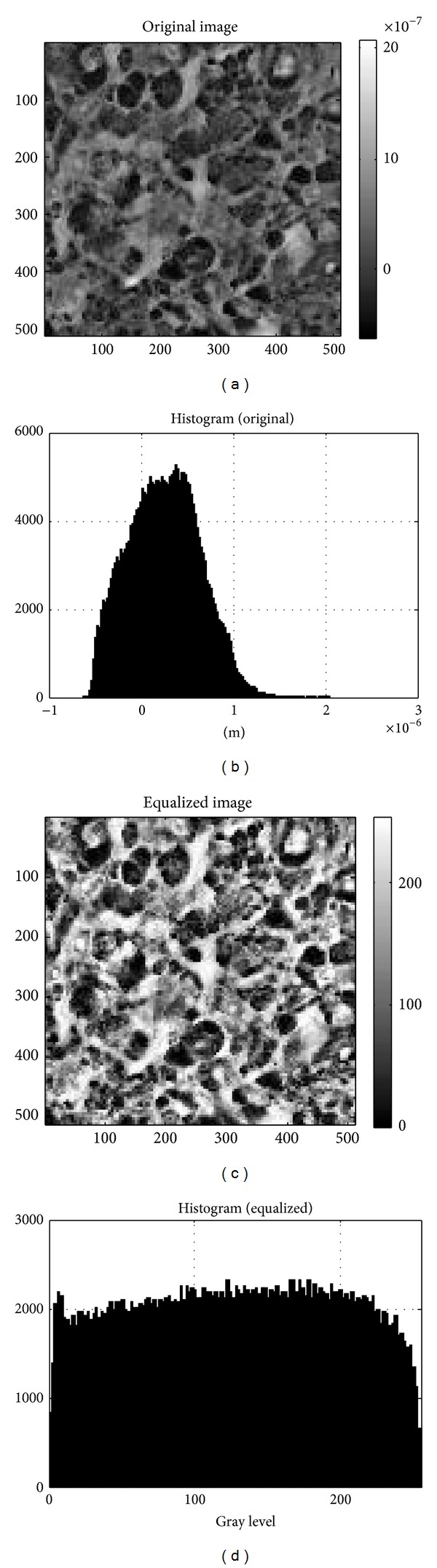
Histogram equalization for a typical glioblastoma multiforme grade IV AFM image. (a) Original AFM image composed of 512 × 512 pixels. The corresponding scan size was 100 × 100 *μ*m^2^. The vertical bar shows differences in height in m. (b) Height histogram of the original data. (c) Equalized AFM image after the transformation step spreading out the height level histogram to a full dynamic range of 256 gray levels. (d) Corresponding equalized histogram.

Figure 1 shows the main processing steps in image analysis. The AFM images were first processed by first order flattening to remove background slope (background correction). As we focus primarily on image structures and spatial correlations (and not so much on absolute image height values), we applied a processing step named “histogram equalization” which increases the global contrast of the image. This is accomplished by a transformation that spreads out the (usually Gauss-curve shaped) height level histograms to a full dynamic range of 256 gray levels. This preprocessing step ensures the comparability of the resulting analysis step. Figures 2 and 3 give the corresponding set of images (original image, original histogram, equalized image, and equalized histogram) according to this process for a typical astrocytoma grade II (Figure 2) and a typical glioblastoma multiforme grade IV (Figure 3). The increase in global contrast can be clearly seen in both types of samples.

**Figure 4:**
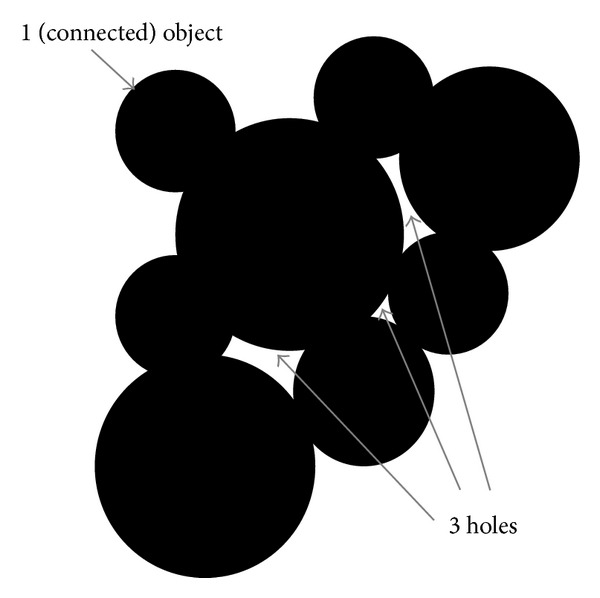
Illustration of the Euler-characteristic. The Euler-characteristic is defined as the total number of objects in the image minus the number of holes in those objects. Exemplarily, the Euler-characteristic of the given binary image is equal to −2.

We finally used Minkowski functionals (or to be more precise “Minkowski measures”)—in particular the Euler-characteristic—as a feature descriptor to characterize global geometric structures related to the topology of the AFM images. The Euler-characteristic is defined as the total number of objects in an image minus the number of holes in those objects.  Figure 4 gives an illustration. Minkowski functionals were first applied in the study of the topology of the density distribution of galaxies in astrophysics [40, 41]. Meanwhile, it is appointed for the quantitative description of complex structures, for example, in medicine (analysis of bone structures in order to improve the diagnosis of osteoporosis [42], X-ray analysis in digital mammography [43]) or materials research. 

In two dimensions, the Minkowski functionals are related to more familiar quantities like the covered image area, the boundary (or contour) length between homogeneous domains, and the Euler-characteristic (i.e., the number difference of connected domains and holes). Here, we focused on Minkowski functionals to characterize the morphology of image domains that result from thresholding AFM height maps at different height levels (i.e., binarization of the AFM image at different gray levels to transform the AFM height map to a stack of level sets).

**Figure 5:**
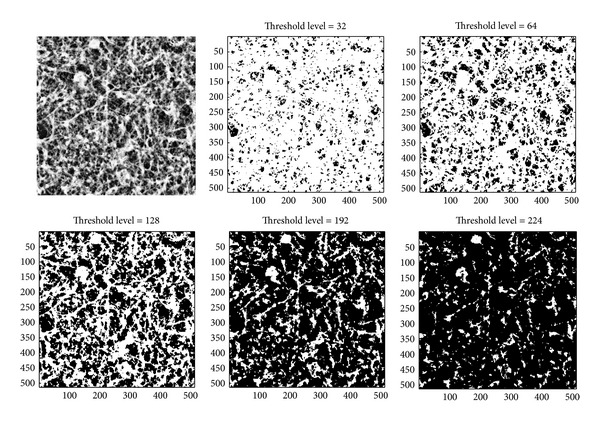
Original AFM image of an astrocytoma grade II, and 5 image examples of binarization corresponding to the height threshold levels 32, 64, 128, 192, and 224.

**Figure 6:**
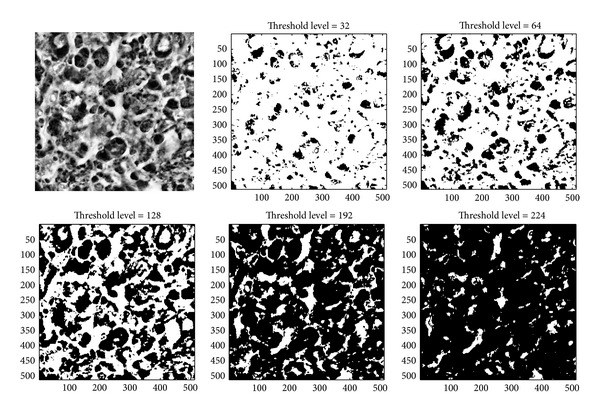
Original AFM image of a glioblastoma multiforme grade IV and 5 image examples of binarization corresponding to the height threshold levels 32, 64, 128, 192, and 224.

Figure 5 shows the original AFM image of an astrocytoma grade II and 5 image examples of binarization corresponding to the threshold levels 32, 64, 128, 192, and 224.  Figure 6 gives the same threshold scheme for a glioblastoma multiforme grade IV. In this way, the Euler-characteristic is an integral geometrical measure that can provide an estimate of the connectivity of a level set structure. This description is topologically invariant (which means it does not change under deformation or scaling) and represents a very compact way to characterize complex image structures.

## 3. Results

### 3.1. Morphological Feature Extraction in AFM Images

**Figure 7:**
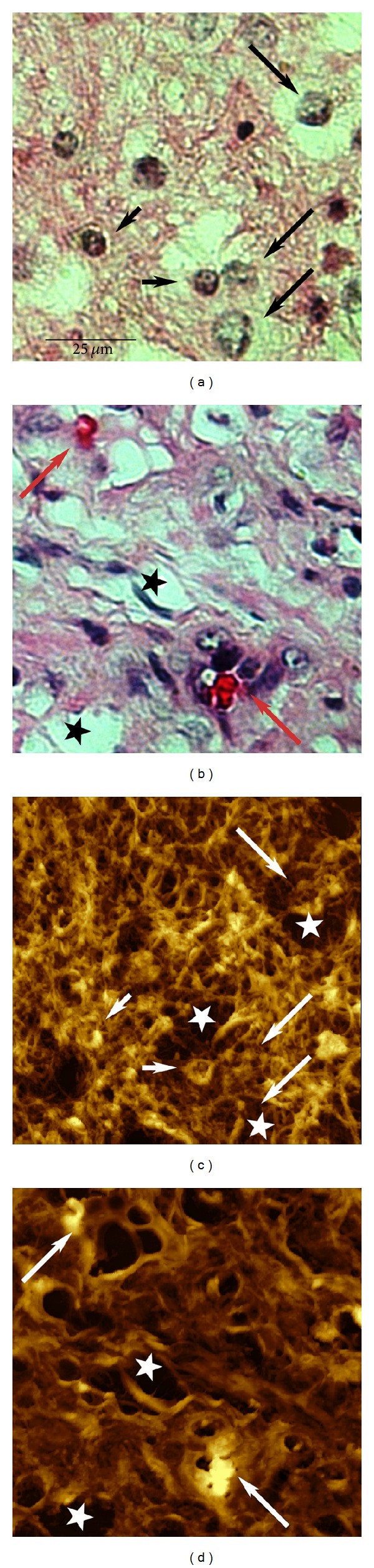
Typical images of brain tumor samples. (a) Light microscopy image of a stained astrocytoma grade II sample at 100x magnification. Healthy microglia (small black arrows), lymphocytes, and tumor cells (large black arrows) can be identified. (b) Light microscopy image of a glioblastoma multiforme grade IV at 100x magnification. The neuropil is highly porous. Most of the polymorphous tumor cells are surrounded by large cavities (black asterisks). Two isolated blood vessels are apparent by erythrocytes and atypical endothelial cells (large red arrows). (c) Corresponding AFM image at 98 × 98 *μ*m scan size of (a). The neuropil as well as many nuclei of healthy cells are well observed (small white arrows). Tumor cells, however, cannot be clearly identified; they are surrounded by large cavities (white asterisks) and blend into the background (large white arrows). (d) Corresponding AFM image at 98 × 98 *μ*m scan size of (b). The tissue is highly degenerated showing irregular large-scaled cavities (white asterisks). Beside two isolated blood vessels no distinct cell features are recognizable.

To prove AFM as a morphological tool in pathology images, brain tumor specimens with light microscopy stained by routine H&E were compared with our AFM results. Typical glioma features like pleomorphic cells or the pseudoglomerulus endothelial proliferation could be recognized very clear in AFM images.  Figure 7(a) is a typical H&E-stained light microscopy image of an astrocytoma grade II with 100x magnification. Microglia and lymphocytes as well as tumor cells can be identified. The nuclei of the healthy microglia cells are regular in shape with a typical size of about 8 *μ*m (small black arrows), whereas the tumorous ones appear large and irregular with a size of up to 20 *μ*m (large black arrows).  Figure 7(b) is an H&E-stained image of a glioblastoma multiforme grade IV with the same magnification. The cells are polymorphous. There are numerous dark areas (black asterisks) up to 25 *μ*m in size. Two isolated blood vessels are also apparent by erythrocytes and atypical endothelial cells (large red arrows).  Figure 7(c) is the corresponding AFM image of the astrocytoma grade II in Figure 7(a). It was taken at a resolution of about 120 nm. Because of the better resolution all relevant morphological features are recognizable. Particularly, eye-catching is the dense network of fine fibres running in all directions. Most of them form junctions with other ones building a well-organized meshwork of bundled strands. This meshwork is called the neuropil. Healthy cell nuclei are also well observed; they appear as raised structures having nearly all the same sizes (small white arrows). The three tumor cells of Figure 7(a), however, cannot be clearly identified; they blend into the background (large white arrows). Tumor cells are often surrounded by large cavities (white asterisks). Their formation is a consequence of retraction artifact, neuropil reduction, or necrosis.  Figure 7(d) is the corresponding AFM image of Figure 7(b). Despite the high resolution only the two isolated blood vessels are well recognized (large white arrows). Distinct cell features, however, are not recognizable like in routine HE samples. As additional morphological feature the fine arrangement of the neuropil is reduced; the tissue appears as a pulpy proliferating mass with irregular large-scaled cavities (white asterisks).

By comparing images of low-grade and high-grade tumors the gradual loss in fine regular anatomy of the neuropil appeared as a noticeable new characteristic tumor feature, because it occurred in close accordance with the tumor type and grading. This gradual loss, which is consistent with a tumor associated loss of functional organization, is accompanied by an increase in neuropil-free areas, which appear dark in the AFM images. Thus, the formation of dark areas was taken as the key feature for the further processing in determining the grading of brain tumors. 

### 3.2. Grade Classification

**Figure 8:**
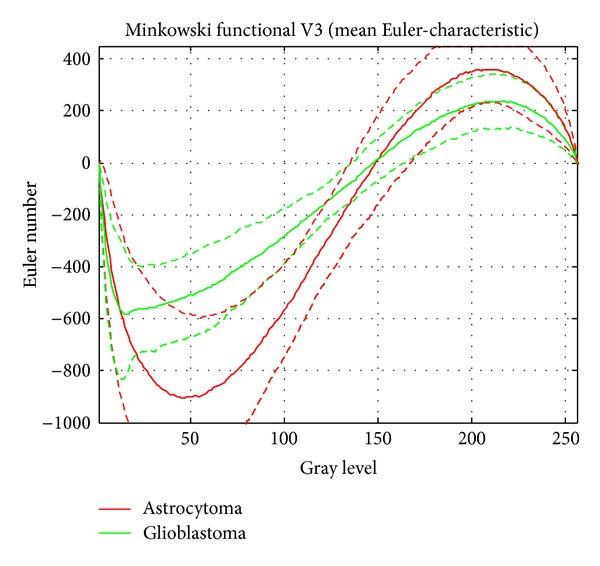
The resulting mean value (solid line) of the Minkowski functional Euler-characteristic and the 1*σ* confidence intervals (dashed lines) for astrocytoma (red) and glioblastoma multiforme (green). Both types of tumors show sigmoid curves which differ in a very characteristic way. The green glioblastoma multiforme curves show an overall flat profile, and the red astrocytoma curves appear more rounded.

Figure 8 shows the resulting mean value (solid line) of the Minkowski functional Euler-characteristic and the 1*σ* confidence intervals (dashed lines) for astrocytomas (red curves) and glioblastoma multiforme (green curves) after having performed the image processing pipeline described in section image analysis. Both types of tumor exhibit sigmoid curves which differ in a very characteristic way in profile. The green glioblastoma multiforme curves show an overall flat profile with a mean value of minimum −582 at gray level 16 and a mean value of maximum 238 at gray level 217. The red astrocytoma curves appear more rounded; their mean value of minimum −905 is shifted to gray level 46 and their mean value of maximum 341 to gray level 210. Not only has the position of the extreme values (minimum, maximum) changed, but also their corresponding Euler-characteristics (*y*-values). At the minimum the mean value reduced by a factor of 1.55 from −582 to −905, whereas at the maximum the mean value increased by a factor of 1.43 from 238 to 341. 

**Figure 9:**
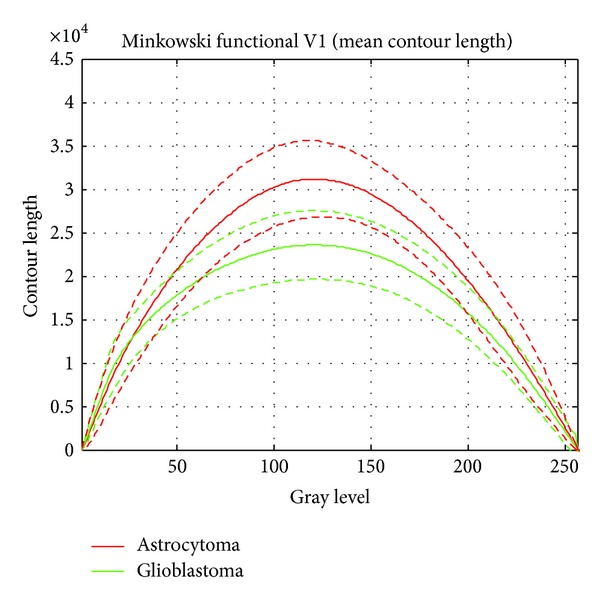
The Minkowski functional contour length and the corresponding 1*σ* confidence intervals (dashed lines) for astrocytoma (red) and glioblastoma multiforme (green). Both curves show a similar parabola-like pattern but differ very characteristically with respect to their mean maximal value.

Another topological descriptor is the Minkowski functional contour length, which is also plotted for both tumor types (astrocytoma: red curves, and glioblastoma multiforme green curves) in Figure 9. Both curves show a similar parabola-like pattern but differ very characteristically with respect to their mean maximal value, which is 2.4 · 10^4^ pixel for glioblastoma multiforme and 3.2 · 10^4^ pixel for astrocytomas.

## 4. Discussion 

An accurate classification of brain tumors is of utmost importance, because it is the basis for an optimal therapy. The search for new grading markers is necessary to improve personalized therapies in a devastating disease like high-grade brain tumors. The WHO has published a classification scheme which is used worldwide for neuropathological typing and grading of brain tumors. The scheme is mainly based on histomorphological features [1], which in case of the great variety of brain tumors and the complexity of morphological features in brain tumors and also within a single tumor are very difficult and subjective. Therefore inter- and intra-observer variabilities of the WHO grading system are high. The literature is full of studies highlighting the lack of reproducibility in evaluating the degree of tumor malignancy [21]. The introduction of exact quantitative methods in image analysis offers the possibility to objectify tumor grading. Recently, a few attempts have been made using light microscopy on routinely stained specimens, and the data are very encouraging [26, 29, 44–50]. As a complete new tool we introduced ultramicroscopic techniques with higher resolution in combination with modern image analysis.

In our study, we used AFM on routine brain tumor samples. Tumor diagnosis and tumor grading were performed by experienced neuropathologists. Artifact-free specimens were selected, showing characteristic tumor features, and the adequate paraffin block was chosen for routine microtome slices 5 *μ*m thick. Then the routine deparaffining was carried on and uncovered tumor slices were measured with the AFM.

The total dataset comprises 113 samples, containing 54 samples of astrocytoma (Grade II) and 59 samples of glioblastoma (Grade IV). Thus, the dataset is nearly balanced which is important for validating the significance of obtained classification accuracies.

For a data-modeling process, creation and selection of appropriate features are essential to the reachable model accuracy. In particular when using image processing for classification, these steps majorly influence the achievable classification results because extracting features from images concern extracting the highest possible amount of information. Here, the given Euler-characteristics provide some very useful data that shall be applied for classification. When using Euler-characteristics directly for classification, for each sample 256 features would need to be considered (i.e., the characteristic's values at 256 gray levels), which gives an unfavorable sample size to dimensionality ratio. However, even if [51] reported that it is possible to derive discriminative models directly out of Euler-characteristics data in such a case, some feature processing shall be applied in order to reduce the dataset's dimensionality. Since according to Figures 8 and 9 different Minkowski functionals (viz. Euler-characteristics and contour length) can be well represented as curves, an obvious step is to reduce the measurements at 256 gray levels to some significant metrics regarding their curves. In this way, [51] presented the usage of 15 distinct geometrical features that have a sufficient descriptive nature for characterizing the Minkowski functionals, such as absolute value and position of extremum points, position of the zero-crossing, steepness measures, or areas under the curves. In this way, a dataset with 113 samples each of 15 features has been obtained.

When aiming at creating generalizable models out of data, the distinction of proper training and test sets is fundamental. Since (as in most biologic applications where measurement costs are high and samples are difficult to obtain) the sample size is very limited, cross-validation is applied for classification. Cross-validation is the method of dividing the available data into *n* subsets, while using *n* − 1 subsets for training, and finally the *n*th subset for testing the model. Therefore, when computing a classification model, its performance can be tested on *n* different configurations of training and test data within the same dataset, while avoiding bias in the evaluation. In this way a significant and generalizable estimate on the model's classification accuracies can be performed even if the sample size is low.

Genetic programming (GP) is an evolutionary algorithm-based method for symbolic classification. It produces interpretable models that allow the assessment of the impact of each single feature in the Minkowski functional curves by this way optimizing the resulting classification accuracy [52, 53]. Analyzing the shape of both, Euler-characteristics and contour length, in all AFM images 15 characteristic features containing discriminative information were identified out of originally 256 ones [51]. Using GP, they were finally computed over all possible parameter configurations. Having performed 100 runs to overcome stochasticity of results, a best model was created that achieved 94.74% classification accuracy.  Table 1 gives the corresponding parameter settings. 

GP's best model resulted in 0.93% better classification accuracy by reducing the dimension *n* of the feature space from 256 to 15. Thus, GP has several advantages. It enables better classification accuracy and works with a manageable number of features that can be extracted from both Minkowski functionals and finally leads to interpretable models. Further information on achievable classification models on this work can be obtained from [51].

A closer view at the Minkowski functional Euler-characteristic revealed a noteworthy, discriminative detail. Between the gray levels 80 and 105, there is a region where the confidence intervals of both mean curves do not intersect. This fact was additionally considered algorithmically using GP and the analysis of this single feature resulted in a prediction accuracy of 89.38%. Obviously, this feature alone has a very high-discriminative capability. Maybe it is a new additional key feature to the conventional grading procedure to separate tumors that are difficult to distinguish, for example, grade II tumors from grade III ones or grade III tumors from grade IV ones. It is also conceivable that this special feature will help in understanding unusual courses of illness. In our analysis, about 10% of the data did not fit the above mentioned prediction accuracy. These data has to be analyzed in the future in a medical orientated paper. Despite the high accuracy of our grading tool the 10% have to be correlated with special tumor features, the tumor region, and finally with the patient history and the tumor outcome. Tumor discovery is mainly based on medical imaging techniques (MRI, CT, or PET), and tumor diagnosis is done by histopathological examination on biopsied or resected tumor tissues. To achieve highest accuracy in classification data mining techniques have been successfully performed in some special cases, especially in MRI and light microscopy images. Reference [36] applied support vector machines (SVM) on MRI images on healthy and brain-tumor-suffering patients. The used algorithm succeeded in classifying all healthy patients and 65% of the tumor-suffering ones. Reference [25] used a special adaptive neurofuzzy interference system based on artificial neural networks and fuzzy logic technique for MRI brain tumor classification. Investigating 4 types of tumors (meningioma, astrocytoma, metastases, and glioma), they resulted in an average classification accuracy of 93.3%. Applied to H&E-stained images, [33] proposed an algorithm based on SVM and least squares mapping. They were able to separate grade II from grade III tumors with a certainty of 97.3% and grade III from grade IV with a certainty of 95.2%, respectively. Taking another model based on fuzzy cognitive maps, [21] achieved a diagnostic output of 90.3% and 93.2% for brain tumors of grade II and grade III, respectively. Another approach was performed by [29] on standardized hematoxylin stained samples performing densitometric analysis of tumor cell nuclei. They compared densitometric features of digitized images with variables of nuclear size, nuclear shape, and proliferation and succeeded in one characteristic feature: the standard deviation of the kurtosis of the gray value histogram which showed a significantly higher value in anaplastic astrocytoma, whereas the other densitometric variables did not give a characteristic hint.

Depending on the used imaging method and the performed data mining technique, classification accuracies between 90.3% and 97.3% were achieved, only taking values concerning the differentiation between different tumor types into account. The proposed AFM-based classification method using GP as classifier achieves 94.74%, which is absolutely comparable with the literature data. AFM has one additional advantage above all concerning the analysis of H&E-stained images. The quality of the images does not depend on the staining protocol, because objects are not viewed, but rather scanned. Thus, variations in color are not detected, which makes AFM insensitive with respect to varying staining conditions and artifacts. 

Imaging histological brain samples using AFM, we were able to discover a new diagnostic feature, the neuropil density, which is a perfect structure for modern computer-based imaging analysis. In combination with data mining techniques this characteristic feature can be used to raise astrocytoma grading to a more objective standard to improve classification accuracy in combination with conventional pathological tumor diagnosis and grading procedure. Additional morphological criteria are of great value to subdivide known tumor entities and to find new grading criteria for therapy decisions. AFM is easily performed on routine pathological samples without additional processing steps. Therefore, the method is easily integrated in the daily routine; AFM could be performed after deparaffination and before the sample staining procedure without diagnostic relay. A further advantage is that AFM is able to analyze sections of old paraffin tumor blocks stored in many pathological labs. Therefore, the method is also useful for retrospective studies on well-defined tumor collections and clinical data.

## 5. Conclusion

Brain tumor classification based on AFM images by using GP has not been done so far. It is a new methodology bringing high-resolution microscopy closer to the clinical practice. It is able to achieve a classification accuracy, which matches or outperforms most of the proposed techniques described in the literature. Additionally, the potential of imaging in the submicron regime enables the characterization of ultrastructures as new diagnostic details within samples in routine pathology that are not visible using conventional medical imaging techniques or light microscopy in tumor diagnostics. AFM is easily implementable in the diagnostic process. Together with data mining techniques, AFM could serve as a powerful new tool in pathological diagnosis and in objectifying morphological features for tumor diagnosis and grading. 

## Figures and Tables

**Table 1:** Parameter settings for best GP result.

Parameter	Tested values
Maximum generations	70, 100
Mutation probability [%]	10, 15
Population size	70
Selector Mutator	Tournament sector Multisymbolic expression tree manipulator: replace branch manipulation, change node type manipulation, full tree shaker, one point shaker
Maximum expression tree depth	8, 10
Maximum expression tree length	25, 50, 80
Symbolic expression tree grammar	Logical operators (the corresponding expression tree symbol set implemented in Heuristic-Lab contains too numerous functions to be mentioned here. Generally, it contains all usually handled logic operators).
